# EVALUATION OF LYMPHATIC SPREAD, VISCERAL METASTASIS AND TUMORAL LOCAL INVASION IN ESOPHAGEAL CARCINOMAS

**DOI:** 10.1590/0102-6720201600040001

**Published:** 2016

**Authors:** Francisco TUSTUMI, Cintia Mayumi Sakurai KIMURA, Flavio Roberto TAKEDA, Rubens Antônio Aissar SALLUM, Ulysses RIBEIRO-JUNIOR, Ivan CECCONELLO

**Affiliations:** 1 Cancer Institute of São Paulo State, São Paulo, SP, Brazil.

**Keywords:** Adenocarcinoma, Squamous cell carcinoma, Neoplasm invasiveness, Lymphatic metastasis, Neoplasm metastasis

## Abstract

**Background:**

Knowing esophageal tumors behavior in relationship to lymph node involvement, distant metastases and local tumor invasion is of paramount importance for the best esophageal tumors management.

**Aim:**

To describe lymph node involvement, distant metastases, and local tumor invasion in esophageal carcinoma, according to tumor topography and histology.

**Methods:**

A total of 444 patients with esophageal squamous cell carcinoma and 105 adenocarcinoma were retrospectively analyzed. They were divided into four groups: adenocarcinoma and squamous cell carcinoma in the three esophageal segments: cervical, middle, and distal. They were compared based on their CT scans at the time of the diagnosis.

**Results:**

Nodal metastasis showed great relationship with of primary tumor site. Lymph nodes of hepatogastric, perigastric and peripancreatic ligaments were mainly affected in distal tumors. Periaortic, interaortocaval and portocaval nodes were more commonly found in distal squamous carcinoma; subcarinal, paratracheal and subaortic nodes in middle; neck chains were more affected in cervical squamous carcinoma. Adenocarcinoma had a higher frequency of peritoneal involvement (11.8%) and liver (24.5%) than squamous cell carcinoma. Considering the local tumor invasion, the more cranial neoplasia, more common squamous invasion of airways, reaching 64.7% in the incidence of cervical tumors. Middle esophageal tumors invade more often aorta (27.6%) and distal esophageal tumors, the pericardium and the right atrium (10.4%).

**Conclusion:**

Esophageal adenocarcinoma and squamous cell carcinoma in different topographies present peculiarities in lymph node involvement, distant metastasis and local tumor invasion. These differences must be taken into account in esophageal cancer patients' care.

## RESUMO

**Racional:**

Conhecer o comportamento das neoplasias esofágicas em relação à disseminação linfonodal, distribuição de metástases e invasão local do tumor, pode auxiliar o manejo dos pacientes.

**Objetivo:**

Descrever o envolvimento linfonodal, disseminação metastática e invasão local dos carcinomas esofágicos, de acordo com a topografia e o tipo histológico do tumor.

**Método:**

Pacientes com diagnóstico de carcinoma espinocelular de esôfago (n=444) e adenocarcinoma de esôfago (n=105) foram retrospectivamente analisados. Foram divididos em quatro grupos: adenocarcinoma e carcinoma espinocelular do segmento cervical, médio e distal. Tais grupos foram comparados baseando-se em tomografias computadorizadas realizadas no momento do diagnóstico.

**Resultados:**

Disseminação linfonodal mostrou grande associação com topografia do tumor. Linfonodos do ligamento hepatogástrico, perigástricos e peripancreáticos foram acometidos principalmente por tumores de esôfago distal; linfonodos periaórticos, interaortocavais, portocavais no carcinoma espinocelular de esôfago distal; e linfonodos subcarinais, paratraqueais, subaórticos nos tumores de esôfago médio. Cadeias cervicais foram acometidas por espinocelulares cervicais. Adenocarcinoma teve maior frequência de acometimento peritoneal (11,8%) e hepático (24,5%) do que carcinoma espinocelular. Considerando invasão tumoral local, quanto mais cranial a neoplasia, mais comum a invasão do espinocelular em vias aéreas, chegando à incidência de 64,7% nos tumores cervicais. Tumores de esôfago médio invadem mais frequentemente aorta (27,6%) e tumores de esôfago distal, o pericárdio e átrio direito (10,4%).

**Conclusões:**

Adenocarcinoma e carcinoma espinocelular de esôfago em diferentes topografias apresentam peculiaridades na disseminação linfática, metástases à distância e invasão local do tumor. Tais diferenças devem ser consideradas no manejo do paciente com carcinoma esofágico.

## INTRODUCTION

Esophageal carcinoma is disease with a high morbimortality rate, even after curative intent surgery^1^ ^,^ ^3^. It turns more noticeable when diagnosis is late - which is quite usual, since more than half of patients is diagnosed in advance stages^5^ ^,^ ^6^. 

Most of its complications are intrinsically associated to the natural history of the disease. Main local complications are associated to local tumor invasion in airway, leading to pulmonary infections or massive hemoptysis. Other complications, such as cholangitis or hepatic failure may be associated with distant disease involvement^6^.

Knowing esophageal tumors behavior, such as lymph-node involvement, distant metastasis and local tumor invasion, is of paramount importance for predicting esophageal cancer clinical manifestations and complications, and so, for the best therapeutic approach.

This study had the objective to describe lymphatic spread in each nodal station; the prevalence and location of distant metastasis; and the prevalence of invaded organs in esophageal carcinoma, according to topography of the tumor and histological type.

## METHODS

The charts of all patients diagnosed with esophageal cancer from January, 2009, to December, 2011, in a single quaternary oncology center (ICESP - Cancer Institute of São Paulo State, São Paulo, SP, Brazil) were retrospectively reviewed. The population studied was composed of 444 patients with squamous cell carcinoma of esophagus (SCC) and 105 patients with esophageal adenocarcinoma (EA). 

To elaborate this research, patients were subdivided into four groups: cervical SCC (15-20 cm from incisors); middle esophagus SCC (comprising upper and middle thoracic tumors, 20-30 cm from incisors); distal esophagus SCC (lower than 30 cm from incisors); and adenocarcinoma of esophagus, based on the first upper GI endoscopy realized in each patient. This classification is in accordance with the 7^th^ Edition of the AJCC Cancer Staging Manual^4^.

Clinical staging was determined at the diagnosis (before any treatment), based on CT scans. Signs of local tumor invasion, lymph-nodal stations with malignant features and distant metastasis were assessed.

### Statistical analysis

Were evaluated absolute and relative frequency by using chi-square test and Likelihood Ratio test - the last one applied just at the impossibility of using chi-square due to a low number of patients studied in a group^2^. Tests were performed with 5% significance level.

## RESULTS

Table 1 summarizes the "T" stage in esophageal cancer TNM stage. The higher SCC topography, the more common was airways invasion (0% in EA, 8.5% in the distal SCC, 46.9% in middle SCC, 64.7% in cervical SCC). Pericardium and right atrium (2.9% in EA; 10.4% in distal SCC; 9.2% in middle SCC; 0% in cervical SCC) were more often invaded in distal SCC. Aorta (0% in EA; 13.2% in distal SCC; 27.6% in middle SCC, 17.6% in cervical SCC) was more often invaded in middle esophageal SCC.

**TABLE 1:** Invaded organs in local advanced esophageal carcinoma

	Esophageal topography and histology					
Local invasion	Cervical SCC	Middle SCC	Distal SCC	EA	Total	p
	(n=51)	(n=239)	(n=106)	(n=105)	(n=501)	
Pancreas	0 (0,0)	1 (0,4)	0 (0,0)	1 (1,0)	2 (0,4)	0,607#
Celiac trunk	0 (0,0)	0 (0,0)	0 (0,0)	4 (3,8)	4 (0,8)	0,006#
Retrocrural space	0 (0,0)	1 (0,4)	3 (2,8)	8 (7,6)	12 (2,4)	0,001#
Gastric fundus	0 (0,0)	1 (0,4)	7 (6,6)	1 (1,0)	9 (1,8)	0,002#
Liver	0 (0,0)	1 (0,4)	1 (0,9)	4 (3,8)	6 (1,2)	0,079#
Pericardium/Heart	0 (0,0)	22 (9,2)	11 (10,4)	3 (2,9)	36 (7,2)	0,003#
Aorta	9 (17,6)	66 (27,6)	14 (13,2)	0 (0,0)	89 (17,8)	<0,001
Cava vein	0 (0,0)	2 (0,8)	0 (0,0)	2 (1,9)	4 (0,8)	0,297#
Brachiocephalic trunk	1 (2,0)	1 (0,4)	0 (0,0)	0 (0,0)	2 (0,4)	0,349#
Airway	33 (64,7)	112 (46,9)	9 (8,5)	0 (0,0)	154 (30,7)	<0,001
Pulmonary hilum	1 (2,0)	10 (4,2)	2 (1,9)	0 (0,0)	13 (2,6)	0,049#
Pleura/Lung	0 (0,0)	6 (2,5)	3 (2,8)	0 (0,0)	9 (1,8)	0,078#
Cervical vessels	3 (5,9)	1 (0,4)	0 (0,0)	0 (0,0)	4 (0,8)	0,013#
Subclavian artery	3 (5,9)	1 (0,4)	0 (0,0)	0 (0,0)	4 (0,8)	0,013#
Vertebral bones/Prevertebral space	4 (7,8)	3 (1,3)	0 (0,0)	0 (0,0)	7 (1,4)	0,004#

Chi-Square test; #=likelihood ratio test; SCC=squamous carcinoma; EA=esophageal adenocarcinoma. The numbers in brackets refer to the percentage.

The lymph-node metastasis showed great relationship with topography of primary tumor site (Table 2). Lymph-nodes of hepatogastric ligament, perigastric and peripancreatic were mainly affected in patients with distal SCC and EA. Periaortic, interaortocaval and at portocaval space lymph-nodes were more commonly found in distal SCC. Subcarinal, paratracheal and subaortic nodes were more common in middle SCC. Neck lymph-nodal stations were more affected by cervical SCC.

**TABLE 2:** Main nodes affected in esophageal carcinoma

	Esophageal topography and histology					
Lymph-nodal metastasis	Cervical SCC	Middle SCC	Distal SCC	EA	Total	p
	(n=50)	(n=224)	(n=102)	(n=91)	(n=467)	
Pre vascular	5 (10,0)	14 (6,3)	5 (4,9)	0 (0,0)	24 (5,1)	0,007#
Retrocrural space	1 (2,0)	6 (2,7)	5 (4,9)	0 (0,0)	12 (2,6)	0,087#
Hepatogastric ligament	3 (6,0)	37 (16,5)	23 (22,5)	28 (30,8)	91 (19,5)	0,002
Peripancreatic	1 (2,0)	4 (1,8)	8 (7,8)	7 (7,7)	20 (4,3)	0,021#
Hepatic hilum	2 (4,0)	3 (1,3)	4 (3,9)	9 (9,9)	18 (3,9)	0,010#
Perigastric	1 (2,0)	15 (6,7)	19 (18,6)	18 (19,8)	53 (11,3)	<0,001
Perispleic	0 (0,0)	3 (1,3)	0 (0,0)	1 (1,1)	4 (0,9)	0,362#
Perigastroesophageal junction	0 (0,0)	2 (0,9)	3 (2,9)	1 (1,1)	6 (1,3)	0,356#
Celiac trunk	3 (6,0)	16 (7,1)	12 (11,8)	11 (12,1)	42 (9,0)	0,325#
Paratracheal	19 (38,0)	95 (42,4)	26 (25,5)	14 (15,4)	154 (33,0)	<0,001
Subcarinal	13 (26,0)	74 (33,0)	16 (15,7)	13 (14,3)	116 (24,8)	<0,001
Pulmonary hilum	4 (8,0)	37 (16,5)	13 (12,7)	13 (14,3)	67 (14,3)	0,438
Subaortic	2 (4,0)	24 (10,7)	5 (4,9)	3 (3,3)	34 (7,3)	0,046#
Periesophagic	22 (44,0)	90 (40,2)	33 (32,4)	36 (39,6)	181 (38,8)	0,465
Cardiophrenic	0 (0,0)	3 (1,3)	1 (1,0)	3 (3,3)	7 (1,5)	0,355#
Supraclavicular	4 (8,0)	18 (8,0)	3 (2,9)	6 (6,6)	31 (6,6)	0,307#
Cervical	13 (26,0)	25 (11,2)	3 (2,9)	6 (6,6)	47 (10,1)	<0,001
Portocava space	0 (0,0)	3 (1,3)	5 (4,9)	0 (0,0)	8 (1,7)	0,027#
Periaortic	3 (6,0)	24 (10,7)	13 (12,7)	0 (0,0)	40 (8,6)	<0,001#
Interaortocaval space	3 (6,0)	12 (5,4)	8 (7,8)	0 (0,0)	23 (4,9)	0,012#
Axillary node/Retropectoral	0 (0,0)	5 (2,2)	0 (0,0)	0 (0,0)	5 (1,1)	0,060#
Aortopulmonary window	1 (2,0)	5 (2,2)	0 (0,0)	0 (0,0)	6 (1,3)	0,091#

Chi-Square test; #=likelihood ratio test; SCC=squamous carcinoma; EA=esophageal adenocarcinoma. The numbers in brackets refer to the percentage.

Concerning distant metastasis (Table 3), peritoneal carcinomatosis (11.8% in EA, 1.7% in distal SCC; 4.2% in middle SCC; 0% in cervical SCC) and liver metastasis (24.5% in EA; 20.3% in distal SCC, 12.5% in middle SCC; 5.3% in cervical SCC) were more common in the EA group compared with the SCC.

**TABLE 3:** Main sites of metastasis in esophageal carcinoma

	Esophageal topography and histology					
Visceral metastasis	Cervical SCC	Middle SCC	Distal SCC	EA	Total	p
	(n=57)	(n=264)	(n=118)	(n=102)	(n=541)	
Pleural Carcinomatosis	0 (0,0)	7 (2,7)	4 (3,4)	3 (2,9)	14 (2,6)	0,345#
Lung	12 (21,1)	38 (14,4)	15 (12,7)	10 (9,8)	75 (13,9)	0,254
Peritoneum	0 (0,0)	11 (4,2)	2 (1,7)	12 (11,8)	25 (4,6)	0,001#
Liver	3 (5,3)	33 (12,5)	24 (20,3)	25 (24,5)	85 (15,7)	0,002
Bones	3 (5,3)	26 (9,8)	8 (6,8)	12 (11,8)	49 (9,1)	0,416
Adrenal	0 (0,0)	5 (1,9)	4 (3,4)	3 (2,9)	12 (2,2)	0,313#
Subcutaneous	0 (0,0)	4 (1,5)	0 (0,0)	3 (2,9)	7 (1,3)	0,100#
Central Nervous System	1 (1,8)	8 (3,0)	2 (1,7)	5 (4,9)	16 (3,0)	0,524#
Kidneys	2 (3,5)	3 (1,1)	1 (0,8)	0 (0,0)	6 (1,1)	0,235#

Chi-Square test; #=likelihood ratio test; SCC=squamous carcinoma; EA=esophageal adenocarcinoma. The numbers in brackets refer to the percentage

## DISCUSSION

Esophageal adenocarcinoma and squamous cell carcinoma in different topographies present peculiarities as lymph-node involvement, distant metastasis and local tumor invasion that must be taken into account in the management of esophageal cancer patients. 

Esophagorespiratory fistula is one of the major complications in SCC patients. It is associated with suppurative pulmonary disorders and hemoptysis, which accounts for some of the main causes of death^3^. In this study, the higher SCC topography, the more common was airways invasion. Under this perspective, when conducting an advanced SCC cancer in a middle or superior topography must be required a special attention for respiratory issues, and earlier use of esophageal prosthesis, when feasible, can be a reasonable choice. 

Lymphatic spread was strongly associated to neoplasm topography and histology. When looking for recurrence on follow-up programs, it should have a high suspicion index for certain lymph-nodes stations, depending on the tumor location and histologic type.

Carcinomatosis was more often found in adenocarcinoma. It should implicate in a more rigorous pre-operative staging program in this patients, mainly in advanced cases, where a pre-operative laparoscopic evaluation should be strongly considered.

## CONCLUSION

Esophageal adenocarcinoma and squamous cell carcinoma in different topographies present peculiarities in lymph node involvement, distant metastasis and local tumor invasion. These differences must be taken into account in esophageal cancer patients' care.
